# Routine Haematological Parameters Associated with HbA1c and Estimated Whole-Blood Viscosity in Diabetes Management: An Exploratory AIC-Based Regression Analysis

**DOI:** 10.3390/jcm15134995

**Published:** 2026-06-26

**Authors:** Jovita I. Mbah, Phillip T. Bwititi, Prajwal Gyawali, Lin K. Ong, Ezekiel U. Nwose

**Affiliations:** 1 School of Health, Medical Sciences & Centre for Health Research, University of Southern Queensland, Toowoomba, QLD 4350, Australia; prajwal.gyawali@unisq.edu.au (P.G.); lin.ong@unisq.edu.au (L.K.O.); 2 School of Dentistry & Medical Sciences, Charles Sturt University, Wagga Wagga, NSW 2678, Australia; pbwititi@csu.edu.au

**Keywords:** haematological parameters, diabetes management, regression, association, glycated haemoglobin, haemoglobin and diabetes

## Abstract

**Background**: Routine full blood count (FBC) testing is part of the haematological workup in diabetes management. There is limited information regarding the contributions of individual haematological parameters to regression models for glycated haemoglobin (HbA1c), estimated whole-blood viscosity (eWBV) and the resulting blood viscosity complications. Importantly, because association and prediction represent distinct concepts, this study extends previous work with a focus on comparative and exploratory relationships. The objective was to compare FBC parameters between higher and lower HbA1c and eWBV groups and identify variables contributing to the Akaike Information Criterion (AIC)-based regression model among diabetics. **Methods**: This laboratory-based mixed quantitative study involved cross-sectional and regression analyses. Fifteen parameters were evaluated, including the following: red blood cell count (RBC) and indices (MCV, MCH, MCHC); platelet count and derived ratios (PRR, PWR, RPR); and white blood cell count (WBC) with lymphocyte ratios (MLR, NLR, PLR). HbA1c and eWBV data were used to create dichotomous subgroups for univariate comparison, followed by exploratory AIC-based model identification of variables. **Results:** HbA1c, RDW, MCV, and RPR, differed significantly between HbA1c groups (*p* < 0.1). Regression analysis identified RDW, MCV, RPR, MCH and RBC as contributors to the HbA1c model. For eWBV, five out of seven parameters (HCT, HB, RBC, WBC, and MLR) showed a significant association. **Conclusions**: These findings highlight haematological parameters with potential values for future predictive model development. Overall, the study supports the usefulness of selected FBC variables as adjuncts in diabetes monitoring with potential utility in understanding glycaemia control and blood viscosity-related complications.

## 1. Introduction

Glycated haemoglobin (HbA1c) is directly linked to diabetes mellitus as it measures the extent of haemoglobin glycation, highlighting the importance of haematology in diabetes management. In the clinical management of diabetes, prophylactic antiplatelet therapy is recommended for primary and secondary prevention of cardiovascular events [1,2], underpinning the importance of thrombosis and haemostasis pathophysiology. Whole-blood viscosity (WBV), the intrinsic resistance to flow, arises from frictional interactions between major blood constituents when blood flows through the vessels [3], and it is an established marker to assess bleeding and thrombosis risks [4]. However, the laboratory testing of WBV has been neglected in clinical practice, something that has been decried [5,6].

It is perhaps pertinent to highlight that the concept of WBV in diabetes should be developed as part of Virchow’s triad, including endothelial dysfunction, hypercoagulability, and stasis in diabetes cardiovascular pathophysiology [7]. The potential basis of stress or oxidative stress per se has been confirmed by evaluating estimated WBV (i.e., eWBV) for over 30 years [8], while the formula and implication for atherosclerosis have also been rearticulated [9].

Although blood cells have emerged as important factors in cardiovascular medicine, they are not included in the guidelines and recommendations for diabetes management [10], perhaps due to a lack of empirical research evidence. In the case of lipid measurements in diabetes management, it is important to acknowledge that there are limitations regarding the accessibility and affordability of the recommended lipid profile tests, especially in low- and middle-income countries [11,12]. These limitations constitute health, economic, and social determinant factors in diabetes management; hence, there is a need to consider cost-effective and common routine haematological measurements. Lipid profiles are established and validated markers, and whole statin therapy relies on the level of LDL and non-HDL cholesterol. However, there is a need for FBC parameters, especially blood cell indices, for the management of coagulopathies and stasis.

It was recently clarified that statistical association and prediction are two different concepts [13,14,15]. However, a systematic review shows that up to 60% of reports erroneously interchange association with prediction [13]. It suffices to say that associations infer cause or effect relationships and must consider confounding factors, whereas prediction analysis neither infers causality nor requires reference to confounders [14].

### 1.1. Statement of the Problem

One study has investigated predictors of haematology parameters among patients with diabetes but did not specifically investigate predictors of HbA1c levels [16]. Therefore, there is a need to evaluate routine FBC parameters in relation to HbA1c and eWBV among patients with diabetes by comparing FBC parameters between higher and lower HbA1c and eWBV groups and to identify FBC parameters that contribute to exploratory AIC-based regression models for HbA1c and eWBV. Studies have also investigated the correlations of haematology parameters with eWBV and HbA1c, including a pilot study on the implications of anaemia [17]. WBV is influenced by red blood cell (RBC) shape, deformity, and levels [4], among other properties, and there is an implicit relationship between HbA1c and cardiovascular disease (CVD) complications.

The problem addressed in this study is underpinned by the fact that all variables constitute confounders. The impact of physiological confounders makes explicit interpretation of variables retained in exploratory regression models for HbA1c and eWBV for an individual variable difficult; hence, evaluation of the significance of each variable is necessary to demonstrate their potential value as candidate markers for future validation. Therefore, this study focuses on investigating the various FBC parameters that contribute to exploratory AIC-based regression models for HbA1c and eWBV.

### 1.2. Research Questions

1. Which FBC parameters differ between higher and lower HbA1c groups?
2. Which FBC parameters differ between higher and lower eWBV groups?
3. Which FBC parameters contribute to an exploratory AIC-based regression model for HbA1c?
4. Which of the FBC parameters contribute to an exploratory AIC-based regression model for eWBV?
5. Hypothesis 1: There are no statistically significant differences in the levels of the FBC parameters between the dichotomous groups of HbA1c.
6. Hypothesis 2: Besides HCT, from which eWBV is calculated, there are no statistically significant differences in the levels of the FBC parameters between the dichotomous groups of eWBV.

## 2. Materials and Methods

*Design*: This was a clinical laboratory-based observational study using a secondary dataset, the same data used in a previously published study [18]. The Akaike Information Criterion regression modelling design was chosen to identify routine haematological parameters that contribute to exploratory AIC-based regression models for HbA1c and eWBV.

*Setting*: Patient laboratory data were collected from a private general practice (Wellness Centre) in Orange, regional NSW, Australia.

*Participants*: Deidentified pathology data of diabetes patients from the general practice were used, and this involved no contact with individual participants.

*Data variables*: Eighteen laboratory variables were collected from the laboratory records, including the following eight independent variables: six FBC parameters—haematocrit, haemoglobin, red cell distribution width (RDW), platelet count, RBC, and WBC— serum total protein levels, and HbA1c.

The remaining ten were dependent variables, comprising eWBV (derived from haematocrit and serum total protein levels, based on published formulae [9,19]) and three indices per blood cell group, namely RBC indices (mean cell volume (MCV), mean cell haemoglobin (MCH), and mean cell haemoglobin concentration (MCHC)) as reported on routine FBC; platelet indices (platelet/RBC ratio (PRR), platelet/WBC ratio (PWR), and RDW-to-platelet ratio (RPR)); and lymphocyte ratios (monocyte-to-lymphocyte ratio (MLR), neutrophil-to-lymphocyte ratio (NLR), and platelet-to-lymphocyte ratio (PLR)).

*Selection criteria*: Inclusion criteria were data that had a full blood count (FBC), as well as HbA1c and serum protein results. The exclusion criterion was incompleteness of data, as previously published [18]. HCT was included in the evaluation of parameters contributing to exploratory AIC-based regression models for HbA1c and eWBV under FBC. Total protein level was not included because it was not part of the routine haematology panel and was unavailable for some participants.

*Study size*: The sample data, which was used in a previous cross-sectional analysis [18], had a size of (N = 244).

*Groupings*: Two subgroupings, namely higher HbA1c and lower HbA1c, were analysed based on dichotomous categorization of data, including higher versus lower subgroupings for eWBV and HbA1c. For the first research question, the dataset was ranked based on HbA1c, and for the second one, the dataset ranking was repeated but based on eWBV levels. In both cases, the upper 50% made up the higher subgroup while the lower half constituted the lower subgroup. Subgrouping reduces statistical power, and findings can therefore only be described as exploratory. The grouping criteria for HbA1c were low (<7.0%) and high (≥7.0%), based on ADA glycaemic control targets. For eWBV, low and high groups were defined using the median split, as no universally accepted clinical thresholds exist; therefore, the lower eWBV group had values < 11.7181, and the higher eWBV group had values ≥ 11.7181.

*Statistical* analysis: Two analyses were performed, both evaluating 15 FBC variables (HCT, Hb, RBC, RDW, WBC, platelet count, MCV, MCH, MCHC, PRR, RPR, PWR, MLR, NLR, and PLR) in relation to HbA1c and eWBV as explained below. Cross-sectional analysis involved the adoption of univariate analyses that compared every FBC parameter between the higher and lower levels of the HbA1c and eWBV groups, respectively. A complete-case approach was used, with no imputation performed. Each analysis used all available observations for the variables included.

The weaker significance was considered in this study, based on the presumption of complex relationships between FBC parameters and HbA1c [20], and the potential impact of multiple comparisons on type 1 error relative to a single multivariate analysis was acknowledged [21]. Hence, weak statistical significance (*p* < 0.1) was set, but standard significance (*p* < 0.05) was also noted. Given the exploratory nature of the work and limited prior evidence on the contribution of routine FBC parameters to HbA1c and eWBV, a slightly more permissive threshold was used at the screening stage to reduce the likelihood of type 2 error and avoid excluding potentially relevant variables. Variable retention in this model was determined using the Akaike Information Criterion, which applies a penalised likelihood approach and therefore provides a more stringent and robust basis for model selection.

Regression analysis employed the Akaike regression model and was performed twice to answer questions 3 and 4 on which FBC parameters contribute to an exploratory AIC- based regression model for HbA1c and eWBV, respectively. A formal power analysis was not performed prior to data collection, as the study used existing data.

*Ethics considerations*: Ethical approval was not required as secondary data were analysed with previous ethics clearance from the Human Research Ethics Committee (HREC) of Charles Sturt University, Australia (re: 2014/158). No primary data were collected from human or animal subjects.

## 3. Results

Descriptive statistics of the cross-sectional data show that the FBC parameters were within the reference interval, and the correlations represented here (Table 1) are as previously reported [18]. However, HbA1c was within the abnormally high range, indicative of poor glycaemic control.

The last two columns of Table 1 show the Pearson correlation coefficients (PCCs) of HbA1c and eWBV. The table shows 17 variables because the serum total protein level was only used to extrapolate eWBV and was not analysed in this study. The results show that, apart from MCV, RDW, and RPR, all FBC parameters were statistically significant for HbA1c (*p* < 0.1), while MCH, RDW, PWR, PRR, NLR, and PLR were statistically significant for eWBV (*p* < 0.1).

The results for the first research question, that is, the comparison of FBC with higher and lower levels of HbA1c, showed that RDW alone achieved a statistically significant difference (*p* < 0.002), while MCV and RPR were also significant at *p* < 0.1 (Table 2). Lower HbA1c and higher HbA1c are dichotomous divisions of HbA1c data, representing the lower 50% and upper 50%, respectively. The statistical test adopted was a cross-sectional univariate analysis. HbA1c*—glycated haemoglobin.

For the second research question on the comparison of FBC with higher and lower levels of eWBV groups, several FBC parameters showed statistically significant differences between the groups (Table 3). For instance, besides the expected trios of HCT, Hb, and RBC, WBC and MLR also showed significant differences. A key finding was that RPR demonstrated significance in association with eWBV, while platelet count did not reach significance. Lower eWBV and higher eWBV are dichotomous divisions of eWBV data, representing the lower 50% and upper 50%, respectively. The statistical test adopted was a cross-sectional univariate analysis (Table 3).

In the analysis of variables retained in an exploratory regression model using *p* = 0.1 as the significance level, the results for the third research question on identifying FBC parameters with contribution to the exploratory AIC-based regression model for HbA1c showed that five parameters were significant contributors to the exploratory AIC-based regression model of diabetic control. These included three that were significantly associated (MCV, RPR, and RDW), as well as MCH and RBC (Table 4). Interestingly, Hgb and HCT were not significant and were considered weaker candidates as markers for future validation. For the fourth research question on identifying FBC parameters with contribution to the exploratory AIC-based regression model for eWBV, seven parameters were significant (Hb, HCT, RBC, MCHC, WBC, MLR, and PRR) and could contribute to the exploratory AIC-based regression model for blood viscosity indicated by eWBV (Table 5).

## 4. Discussion

This study is an extension of a previous report [18]. Both studies are based on the same dataset and examined how the same FBC parameters are related (i.e., associated or correlated) with glycated haemoglobin (HbA1c) and estimated whole-blood viscosity (eWBV). However, they adopt different emphases and designs. The previous evaluation of relationships was based on a periodic cohort study design and made comparisons between periodic groups of the cohort, with each group comprising the same *n* = 244, whereas the present study uses cross-sectional data categorised into dichotomous groups, with each group comprising *n* = 122. In the previous report, relationships were also investigated through correlation analysis, which provides an indication of negative or positive relations, shown here in Table 1, whereas the present study uses inferential statistics (that is, *p*-values) for comparison between dichotomous groups.

Table 2 shows that only RDW is significantly different (*p* < 0.002). Although platelet count showed no significance, when RDW was incorporated as the RDW-to-platelet ratio, this led to statistical significance (*p* < 0.084). Among the common RBC indices, MCV also showed statistical significance (*p* < 0.072), but not haemoglobin level. This suggests that erythrocyte variables, notably RDW and MCV, may be most associated with HbA1c. Further, the first hypothesis is rejected; hence, it is submitted that changes in levels of the FBC parameters affect HbA1c.

RDW has been shown to be a prognostic marker to gauge DM complications and mortality risk, including the risk of developing DM, which is associated with a low RDW [22]. There are indications that in people with diabetes who have a low level of RDW, the latter may be influenced by DM duration, haemoglobin level, and eGFR [23]. Likewise, MCV has been shown to be associated with DM, and RDW and MCV are positively associated with HbA1c levels [22,24]. However, there are studies that differ from the observations reported here. For instance, the observation of non-significance in PWR is not supported by a report suggesting that an increased PWR level is significantly correlated with low diabetes risk [25]. The implication and significance of the present report lie in the use of available routine laboratory medicine services, especially when interpreting HbA1c for diabetes management, i.e., when the result of HbA1c is not conclusive [20], the RDW, RPR, and MCV from routine FBC are available for review at no further cost.

Table 3 shows that WBC and platelet count levels, as well as RPR and MLR, are statistically significantly different. The eWBV is extrapolated from HCT; therefore, the significance regarding RBC and Hb is considered unremarkable. However, none of the RBC indices showed statistical significance, a notable finding suggesting that besides erythrocyte variables, other blood cell counts are associated with eWBV. Therefore, the second hypothesis is rejected; hence, it is suggested that changes in levels of the blood cell counts affect eWBV.

It has been reported that the impact of leucocytosis and thrombocytosis on blood viscosity underpins the concepts of leukapheresis and thrombopheresis, respectively [26,27,28], and the observations reported here agree with previous validation studies [29]. The implications and significance of this report lie in the use of available routine laboratory medicine services, especially when considering blood flow issues such as bleeding or thrombosis for diabetes management. For instance, evaluation of bleeding risk in diabetes would benefit from a combination of eWBV and platelet count [30,31].

Table 4 shows that the haematological variables retained in exploratory regression models for HbA1c are MCV, MCH, RDW, RPR, and RBC at the weak statistical significance level of *p* < 0.1, among which three are significantly associated (Table 2), and two are non-significantly associated.

At least two inferences can be drawn from the observations. First, regarding haemoglobin molecules, instead of focusing on Hb or MCHC levels to enhance glycation in diabetes, it is the volume (MCV) and weight (MCH) that are relevant. In addition to these two parameters, two others (RBC and RDW) related to the blood cells carrying Hb are variables retained in the exploratory regression model. The second inference relates to platelets: RPR, but not platelet count, is a variable retained in the exploratory regression model. Thirdly, 2 out of 12 (16.67%) of the non-significant parameters are variables retained in the exploratory regression model.

There is a dearth of studies on haematological predictors of HbA1c. One study indicated that age and gender, as well as comorbidity, are key predictors for haematological parameters [16]. However, it did not investigate how or if the haematological parameters may predict HbA1c. Another study investigated the prediction of DM using machine learning and reported WBC as the main haematological parameter [32].

A significant observation from this study is the implication of diabetic anisocytosis (high RDW) as well as iron deficiency anaemia (low MCV). Notably, it is often observed that low RDW is seen in hyperglycaemia and prediabetes, but in contrast, high RDW positively associates with HbA1c [33]. Therefore, the possibility that low RDW could be negatively associated with HbA1c in established diabetes has not previously been addressed, possibly due to limited data. The significance of this discourse lies in reviewing the prognostic value of RDW for diabetes management.

Table 5 shows the observed variables retained in the exploratory AIC-based regression model for eWBV and demonstrates that 7 out of 15 parameters (HCT, Hb, RBC, MCHC, WBC, MLR, and PRR in descending order of strength) could contribute to exploratory regression for blood viscosity in diabetes. Among these seven parameters, two (MCHC and PRR) are non-significantly associated (Table 3), while another two (RPR and platelet count) are significantly associated and non-contributors to exploratory regression (Table 5).

Three inferences can be made. First, aside from HCT, which is used in the eWBV algorithm, and RBC, which indicates polycythaemia, the Hb level can also independently be retained as a variable in an exploratory regression model for eWBV. Second, the WBC, which may indicate leucocytosis, is a contributor to the exploratory regression model for eWBV; third, PRR, but not platelet count, is a contributor to the exploratory regression model.

A study in Pakistan reported that haematological parameters, including haematocrit, NLR, and PLR, may be predictors of diabetic microvascular complications [34], which appears to differ from the present observation. However, statistical analyses in that study were limited to the investigation of potential associations and comparisons between diabetes and apparently healthy individuals, constituting an example where association and prediction may have been used interchangeably. Another report postulates that RPR is a contributor to an exploratory model for haemorrhage, that is, low blood viscosity [35], but the study did not focus on diabetes. The present report focuses on diabetes and indicates an association but not a contributor to the exploratory regression model, thus presenting a different perspective on haematological parameters that are retained in exploratory regression for blood viscosity in diabetes. Association tests evaluate mean differences between groups, while AIC regression evaluates the contribution of variables to model fit, accounting for multivariable interactions. A variable may not differ significantly between groups but may still improve the overall explanatory power of the regression model.

The implication of this observation is the contribution of empirical data on association versus contributors to the exploratory regression model to blood viscosity for clinical CVD management in diabetes, which is significant in preventive medicine. Further, RPR has been reported as a clinical parameter associated with thrombosis and as a contributor to exploratory regression of haemorrhage [35]. Thus, this report presents laboratory-based evidence of routine haematological parameters that can be used alongside eWBV testing for clinical decision making on issues such as erythropheresis and leukapheresis, for which there are currently no prognostic tests. The study did not develop or validate a clinical prediction model, and findings represent exploratory identification of FBC parameters retained in AIC-based regression, not a validated biomarker.

### 4.1. Significance of the Study

The study shows that RDW, MCV, and RPR are associated with and are contributors to exploratory regression for HbA1c whereas MCH and RBC may not be associated with HbA1c (Table 2) but are contributors to exploratory regression to its changes (Table 4); HCT, Hb, and RBC are associated with, and are contributors to exploratory regression, for eWBV; and MCHC, WBC, MLR, and PRR may not be associated with (Table 3), but are contributors to exploratory regression to changes in eWBV (Table 5).

It is known that age and gender are confounding factors in the correlation between HbA1c and haemoglobin [36]. It is also reported that clinical laboratories play a critical role in the healthcare of individuals with diabetes [37], which indeed underpins the established concept of laboratory medicine [38]. A point that needs clarification is the inverse relationship between HbA1c and Hb arising from glycation, possibly reducing Hb levels as a result of increased RBC fragility in hyperglycaemia [39]. This decrease in Hb can trigger a physiological response in erythropoiesis, creating potential correlations with red blood cell (RBC) count and indices [40].

The primary significance of this report lies in advancing the potential of utilising haematological tests as a contributor to diabetes management, especially where access to lipid profile measurement is limited. The goal is not to replace HbA1c but to explore whether FBC parameters may serve as adjunct indicators in resource-limited settings, as early markers of haematological or viscosity-related complications. Hence, the findings are preliminary, hypothesis-generating, and not practice-changing. It is neither evidence of a validated prediction tool nor demonstrated clinical predictive value. The utilisation of haematological parameters is cost-effective in the management of diabetic complications; accessible, especially for people in low-income countries; and could contribute to the early detection of complications in diabetes mellitus, as well as identification of people with diabetes who are at risk of anaemia [34,40].

Further applications include diabetes self-management programmes and health promotion campaigns, as well as further research on diabetes management. Moreover, haematological parameters could be used not only to predict cardiovascular risk in diabetes but also to monitor glycaemic control [32].

The previous report emphasised the pattern and relative strength of correlations, as well as the superiority of RBC indices over platelets and lymphocyte ratios in association with HbA1c and eWBV [18]. The present study emphasises the variables contributing to exploratory AIC-based regression of FBC parameters to HbA1c and eWBV. Together, the studies demonstrated a consistent and logical reason to embrace the concept that routine FBC parameters, especially RBC indices, could be low-cost adjunct markers in diabetes management, particularly for assessing blood viscosity-related risks.

### 4.2. Limitations

The study was limited to secondary archived pathology data. The detailed medical history of the participants is unknown, such as comorbidities or whether participants were on medications at the time of testing. It is also worth mentioning that this limitation is the focus of another project within the larger research programme. HbA1c may be physiologically influenced in iron deficiency, which may partially affect the observed associations; unfortunately, iron results were unavailable in the dataset, and future studies should incorporate ferritin, transferrin saturation, and haemoglobinopathy screening. There is an absence of covariates such as demographic variables and nutritional or haematinic status, which may have influenced the haematological parameters in this study. Findings should therefore be interpreted as preliminary and hypothesis-generating rather than adjusted causal estimates. Further studies should incorporate covariates such as clinical history, demographic variables, comorbidity profiles, medication use, and nutritional or haematinic status. Subgrouping reduces statistical power; therefore, findings should be described as exploratory, and larger prospective-powered studies are recommended. Age and gender influence haematology in diabetes, and these covariates were also unavailable, which constitutes a limitation. Results should therefore be interpreted cautiously and validated in datasets with demographic adjustments.

Additionally, the study is an early step toward prediction-model development, not a completed prediction-model study. Clinical prediction would require formal model development and validation, including prespecified prediction timing, prediction horizon, model performance assessment, calibration, discrimination, internal validation, external validation, and assessment of clinical utility. This study focuses on advancing clinical utility or routine haematological parameters in managing cardiovascular complications in diabetes. We acknowledge other measures of glycaemia, such as fructosamine, glycated-albumin and continuous glucose monitoring (CGM). However, fructosamine and glycated albumin are neither routine clinical pathology tests nor accessible in resource-limited health services. Therefore, while continuous glucose monitoring is encouraged, this report advances additional clinical utility of routine haematological parameters in diabetes management, especially with clinical relevance of haemorrheology complications; therefore, other measures of glycaemia, such as fructosamine, glycated-albumin and continuous glucose monitoring, were not considered.

## 5. Conclusions

This study shows that RDW, MCV, and RPR are associated with and contribute to an exploratory AIC-based regression model for HbA1c, while MCH and RBC are only contributors to an exploratory regression to changes in HbA1c. Further, HCT, Hb, and RBC are associated with and are contributors to exploratory regression to eWBV, while MCHC, WBC, MLR, and PRR may not be associated with but are contributors to exploratory regression to changes in eWBV. The observed differences in FBC parameters between higher and lower eWBV groups highlight a pathophysiological framework consistent with heightened vulnerability to cardiovascular risk and diabetes complications.

The primary advantage of this study lies in advancing the potential of utilising haematological tests in diabetes management, especially where access to lipid profile measurement is limited. These tests could also be utilised as a rapid indicator for the early detection of complications in diabetes mellitus, as well as for identifying people with diabetes who are at risk of anaemia.

They could also be applied in diabetes self-management programmes, health promotion campaigns, and further research on diabetes management. Further longitudinal and outcome-based investigations are essential, as the results in this study are exploratory and FBC parameters are valuable as accessible adjuncts rather than a standalone predictive tool. No clinical endpoints or longitudinal outcomes were assessed; therefore, clinical applicability remains speculative.

### Exploratory Nature of Study/Appropriate Interpretation

These findings should be interpreted as exploratory evidence identifying candidate haematological variables for future prediction model development. They do not establish a validated clinical prediction model or demonstrate that the identified variables have independent clinical predictive utility.

## Figures and Tables

**Table 1:** Correlation analysis outcome of parameters as previously published [18].

Parameters	Averages	HbA1c* (PCC)	eWBV* (PCC)
HbA1c %	7.67	1.000	
eWBV mPas	17.18	−0.019	1.000
Hgb g/L	148.01	0.027	0.463
RBC 10^6^/µL	5.02	0.087	0.524
HCT %	0.45	−0.014	0.491
MCV (fL)	88.91	−0.192	−0.135
MCH (pg)	29.55	−0.099	−0.023
MCHC (g/L)	332.55	0.102 **	0.146
RDW %	13.21	−0.159	−0.044
WBC 10^3^/L	7.85	0.049	0.223
Platelets 10^3^/µL	264.66	0.060	0.113
PWR	35.60	−0.036	−0.086
PRR ^†^	1:20.10	0.007	−0.078
RPR	20.11	0.111	0.142
MLR	0.41	−0.044	0.139
NLR	1.94	0.063	0.093
PLR	117.66	0.010	−0.028

N = 244. Cross-sectional data evaluated to visualise how parameters correlate with eWBV and HbA1c. HbA1c*—glycated haemoglobin, eWBV*—estimated whole-blood viscosity, mPas—millipascal-second, PRR ^†^—number of RBCs per platelet count. The cross-sectional adopted univariate analysis produced correlations; ** approximated to *p* = 0.1.

**Table 2:** Association of FBC parameters with HbA1c*.

Parameters	Lower HbA1c*	Higher HbA1c*	*p* Values
HbA1c %	6.212	9.055	0.000 *
eWBV mPas	17.250	17.110	0.281
Hgb g/L	148.000	148.000	0.834
RBC 10^6^/µL	5.000	5.000	0.700
HCT %	0.450	0.440	0.506
MCV (fL)	89.400	88.400	0.072
MCH (pg)	29.700	29.400	0.364
MCHC (g/L)	331.700	333.300	0.286
RDW %	13.500	13.000	0.002 **
WBC 10^3^/L	7.950	7.750	0.500
Platelets 10^3^/µL	261.324	267.815	0.477
PWR	34.949	36.221	0.401
PRR	20.257	19.958	0.673
RPR	19.502	20.687	0.084 *
MLR	0.552	0.272	0.862
NLR	1.890	1.990	0.413
PLR	113.380	121.700	0.289

HbA1c*—glycated haemoglobin. * Significant at *p* < 0.1. ** Significant at *p* < 0.05.

**Table 3:** Association of FBC parameters with eWBV*.

Parameters	Lower eWBV	Higher eWBV	*p* Values
HbA1c %	7.675	7.680	0.991
eWBV mPas	16.440	17.900	0.000 **
Hgb g/L	143.000	153.000	0.000 **
RBC 10^6^/µL	4.800	5.200	0.000 **
HCT %	0.430	0.460	0.000 **
MCV (fL)	89.300	88.400	0.130
MCH (pg)	29.600	29.500	0.693
MCHC (g/L)	331.400	333.600	0.137
RDW %	13.300	13.200	0.528
WBC 10^3^/L	7.380	8.280	0.002 **
Platelets 10^3^/µL	256.552	270.992	0.077 *
PWR	36.530	34.669	0.224
PRR	19.795	20.533	0.389
RPR	19.363	20.731	0.030 **
MLR	0.271	0.546	0.001 **
NLR	1.860	2.010	0.189
PLR	118.320	116.630	0.266

FBC—full blood count, eWBV*—estimated whole-blood viscosity. * Significant at *p* < 0.1. ** Significant at *p* < 0.05.

**Table 4:** Summary of Akaike regression for HbA1c.

Parameter	AIC*	*p* Values
MCV (fL)	816.5300	0.0024 **
MCH (pg)	821.5901	0.0378 **
RDW %	821.9111	0.0468 **
RPR	822.0910	0.0512 *
RBC 10^6^/µL	823.3128	0.1011 ***
Platelet 10^3^/µL	824.0448	0.1711
PLR	824.4816	0.2271
NLR	824.6719	0.2635
MCHC (g/L)	825.3638	0.4504
PWR	825.7456	0.6587
MLR	825.8761	0.8022
HCT %	825.8919	0.8274
Hgb g/L	825.9126	0.8701
PRR	825.9239	0.9011
WBC 10^3^/L	825.9388	0.9784

AIC*—Akaike Information Criterion. * Significant at *p* < 0.1. ** Significant at *p* < 0.05. *** Approximated to *p* = 0.1.

**Table 5:** Summary of Akaike regression for eWBV.

Parameters	AIC*	*p* Value
HCT %	426.0169	0.0000 **
Hgb g/L	431.6228	0.0000 **
RBC 10^6^/µL	433.4218	0.0000 **
MCHC (g/L)	494.5025	0.0281 **
WBC 10^3^/L	495.3056	0.0409 **
MLR	496.1439	0.0725 *
PRR	496.6806	0.1003 ***
NLR	497.5972	0.1795
RPR	497.6831	0.1869
Platelet 10^3^/µL	498.5702	0.3545
MCHC	498.6814	0.3844
PWR	498.6994	0.3970
RDW %	498.9590	0.4975
MCV (fL)	499.2620	0.6792
PLR	499.4200	0.9399

AIC*—Akaike Information Criterion. * Significant at *p* < 0.1. ** Significant at *p* < 0.05. *** Approximated to *p* = 0.1.

## Data Availability

Data presented in this study can be found here https://doi.org/10.6084/m9.figshare.30904331.
